# *KIT* exon 10 variant (c.1621 A > C) single nucleotide polymorphism as predictor of GIST patient outcome

**DOI:** 10.1186/s12885-015-1817-5

**Published:** 2015-10-24

**Authors:** Mehdi Brahmi, Laurent Alberti, Armelle Dufresne, Isabelle Ray-Coquard, Philippe Cassier, Pierre Meeus, Anne-Valérie Decouvelaere, Dominique Ranchère-Vince, Jean-Yves Blay

**Affiliations:** 1 Department of medical oncology, Centre Leon Berard, 28 rue Laennec, Lyon, France; 2 Cancer Research Center of Lyon, INSERM UMR 1052, CNRS UMR 5286, Centre Leon Berard, 28 rue Laennec, Lyon, France; 3 Pathology Department, Centre Leon Berard, 28 rue Laennec, Lyon, France

**Keywords:** GIST, KIT, Single nucleotide polymorphism, Prognostic factor

## Abstract

**Background:**

Tumor genotype plays a crucial role in clinical management of GIST. Whether genetic polymorphism of *KIT* may influence GIST patient outcome is unclear.

**Methods:**

We investigated the biological and clinical significance of the presence of *KIT* exon 10 variant (c.1621 A > C), *KIT*^*L541*^, in a transfected cell line (3 T3 L541) and in two retrospectively collected series of 109 GIST patients in total. The control group consisted of 60 healthy donors collected at the French department of blood transfusion.

**Results:**

In the 3 T3 L541 cell line, KIT^L541^ protein exhibited a spontaneous phosphorylation status comparable to that of wild-type KIT but displayed a phosphorylation pattern of AKT and ERK1/2 that was found similar to that of the classical mutated forms of the KIT receptor. Of 109 patients enrolled in this retrospective translational research study, 24 (22 %) harboured *KIT*^*L541*^, similarly to the control group of healthy donors (*n* = 10 of 60, 17 %). A higher prevalence of the variant *KIT*^*L541*^ was observed in patients with metastatic status at diagnosis (*KIT*^*L541*^ correlated nine of 22 versus 15 of 87, *p* = 0.02). In addition, patients with *KIT*^*L541*^ and localized GIST had a higher rate of relapse at 5 years and lower relapse free survival at 5 years in univariate, as well as in multivariate analysis. Response rate and duration of response to imatinib was similar in *KIT*^*L541*^ and *KIT*^*M541*^ patients.

**Conclusion:**

*KIT*^*L541*^ genotype is associated with a higher risk of metastasis at diagnosis and a higher risk of relapse in GIST patients.

**Electronic supplementary material:**

The online version of this article (doi:10.1186/s12885-015-1817-5) contains supplementary material, which is available to authorized users.

## Background

GIST (gastrointestinal stromal tumors) are the most common mesenchymal tumors of gastro-intestinal tract and account for 15 to 20 % of soft tissue sarcomas and 1 to 3 % of all malignant digestive tumors [1]. Their annual incidence is approximately 12–15 cases per million inhabitants [2, 3]. GIST are paradigmatic models of cancers with a driver mutation of an oncogene. These driver mutations may occur in different genes but are mutually exclusive: most affect the proto-oncogenes *KIT* (75-80 %) and *PDGFRA* (5–10 %), which encode for mutated proteins with ligand independent constitutive activation of tyrosine kinase receptor. There are a variety of *KIT* and *PDGFRA* mutations, on different portions of the receptors. These different mutations are associated with different risk of relapse after resection of the primary tumor but also impact on the activity of imatinib (selective inhibitor of KIT and PDGFR-α) [4–6].

Tumor genotype plays a crucial role in clinical management of GIST and should now be recommended as a standard procedure when possible, according to ESMO guidelines [7]. Beyond these driver mutations, additional factors such as single nucleotide polymorphism (SNP) and epigenetic phenomena were found to be associated with GIST patient outcome [2, 8–12], suggesting the importance of somatic genome.

A SNP of *KIT* exon ten, encoding for the substitution of a methionine in position 541 by a leucine (*KIT*^*L541*^) in the mature KIT protein, has already been reported to be associated with several diseases, including aggressive fibromatosis (AF) [13–15], chronic myelogenous leukemia (CML) [16], pediatric mastocytosis [17] and more recently in chronic eosinophilic leukemia [18]. The potential impact of this variant, observed in about 20 % of the population, on GIST biology, clinical presentation and response to treatment has never been explored.

In the present work, the clinical and biological significance of the presence of the variant protein in GIST was investigated using two approaches: first exploring KIT^L541^ signalling in transfected cell lines and then exploring the prevalence and prognostic value of *KIT*^*L541*^ in GIST patients, investigating 2 distinct series of patients.

## Methods

### Transfected NIH3T3 cell lines

Murine fibroblast cell lines (NIH3T3) were stably infected with oncoviral vectors containing the full-length human *KIT* cDNA either 1) wild-type (**WT**) with M541, 2) encoding for canonical GIST mutations (del557-558 = **D6** or del564-581 = **D54**), 3) encoding for both WT KIT and canonical mutations (**WT/D6** or **WT/D54**), 4) encoding for homozygous polymorphic *KIT* (1621 A > C = **L541**), or 5) with the empty vector (**MIGR**) [19]. The stably infected NIH3T3 cells expressed the KIT protein in two forms: the mature and the immature proteins of 145 and 125 kDa, respectively [19].

### Cell culture

Cells were cultured at 37 °C humidified atmosphere containing 5 % CO2 in DMEM with 10 % newborn calf serum (Life Technologies), 2 % penicillin/streptomycin, 1 mM L-Glu. For transfected cells, medium was supplemented with 1/1000 G418 (Invitrogen) to maintain a selective pressure for vectors’ expression. Fifty ng/mL of Chinese hamster ovary–mouse rhSCF (recombinant human Stem Cell Factor, R&D systems) were added for cells expressing WT *KIT*, so that both the expression and the activation state of WT and mutant forms of *KIT* were equivalent. Treated cells medium was supplemented with 1 μM of Imatinib (Novartis) 24 h before lysis.

### Protein extraction and Western blotting

Cells were lysed in Petri plate using RIPA buffer (10 mM Tris pH 7.4, 150 mM NaCl, Triton 1 %, DOC 0.5 %, SDS 0,1 %, EDTA 1 mM, 1 mM ortho vanadate) plus protein inhibitors 30 min at 4 °C. Proteins were quantified using a Dc protein assay kit (Biorad®). Migration was performed using gradient gel (Invitrogen® NuPAGE® 4–12 % Bis Tris Midi Gel, 1.0 mm) in NuPAGE® MES SDS running buffer (Invitrogen®). After electrophoretic separation, proteins were electrotransferred on a polyvinylidene difluoride membrane (Millipore®). The membrane was then blocked for 1 h at room temperature with ECL™ Advance Blocking Agent 0.2 % (GE healthcare Limited®) in TBS/Tween 0.1 % and primary antibody incubated for one night at 4 °C. The membrane was washed three times in TBST 0.1 %, and secondary antibodies were incubated for 1 h at room temperature. Antibodies characteristics are presented in Additional file 1: Table S1 online. After washing, revelation was performed using Lumigen™ TMA-6 reagents (GE healthcare Limited®) and Amersham Hyperfilm™ ECL (GE healthcare Limited®).

### Tumor and blood samples

This study was performed as a retrospective translational research program on tumor and blood samples of 109 GIST patients. The first series included 87 resected primary GIST untreated until recurrence and the second series included 22 metastatic or locally advanced/unresectable at diagnosis GIST. A control group consisted of 60 blood samples from healthy donors collected at the French department of blood transfusion.

All samples of the 109 patients were obtained from pathology department:

- Paraffin-embedded tissues samples of 98 GIST, at initial diagnosis, obtained by biopsy or surgical excision to establish the diagnosis of the disease
  - 44 included in “EMS” project [20] from February 2006 to July 2007
  - 35 patients “off-protocol” treated in Centre Léon Bérard from May 2004 to June 2012
  - 19 patients included in “BFR14” trial [21] from June 2002 to April 2006 (15 patients in whom dry pellet PBMC corresponding were available)
- Dry pellets of peripheral blood mononuclear cells (PBMC) frozen of 11 patients included in “BFR14” trial.

All patients signed informed consent to participate to research according to the French laws. Of note, for patients included in the BFR14 study, the protocol and its connected informed consent form (ICF) were reviewed and validated by a national ethics committee (CPP Sud-Est IV); for the others patients, an Institutional Review Board (The Centre Léon Bérard Clinical Trial Review Committee) reviewed and agreed with the study protocol and the ICF.

### DNA/RNA extraction

Total DNA and RNA was extracted from tumors and PBMCs, using *QIAmp DNA FFPE Kit*, *QIAamp RNeasy FFPE Kit* and *Universal Kit* (Qiagen®, France) according to the manufacturer’s instructions, and quantified by spectrophotometry (NanoDrop™ ND-100 instrument, Thermo Fisher Scientific, Waltham, MA). After several washing with toluene and ethanol, FFPE tumors were lysed using ATL buffer and proteinase K and incubating at 56 °C for 24–48 h and 90 °C 1 h. DNA and RNA extraction was carried out using *QIampMiniElute Spin Column* and *RNeasy MiniElute Spin Column.*

PBMCs were lysed using RLT buffer and proteinase K. DNA and RNA extraction was carried out using *AllPrep DNA spin column* and *Rneasy Mini spin column.*

### Competitive Allele-Specific Taqman® PCR (CAST®-PCR) and sequencing

The detection of the SNP was performed by Competitive Allele-Specific Taqman® PCR technology provided by Applied Biosystems®, using the method extensively described by Dufresne et al. [15]. We both performed a CAST®-PCR and a classical sequencing in 13 cases. Polymerase chain reaction (PCR) was used to amplify *KIT* exon 10. The primers used were designed by Primer Express and generated by Beckman Coulter Genomics: 5’-GTA-ATC-GTA-GCT-GGC-ATG-ATG-TG-3’ (sense) and 3’-AAC-CAT-TTA-TTT-GTT-CTC-TCT-CCA-GAG-5’ (antisense). PCR was carried out using Taq DNA polymerase (Transgenomic, Omaha, NE, USA) with the following cycle conditions: denaturation at 95 °C, followed by 10 cycles at decreasing temperatures between 66 and 61 °C with a decrement of 0.5 °C per cycle, and additional extension at 72 °C. The primers used for sequencing were the same as those used for amplification. Before sequencing, PCR products were purified using Centriprep centrifugal filter device (Millipore, Billerica, MA, USA). The purified PCR product was then subjected to automated sequencing performed by Beckman Coulter Genomics. Results of that double detection of the SNP, by sequencing and CAST®-PCR, were always consistent.

### Statistical analysis

Statistics were performed using SPSS statistical software (version 15.0 for Windows, SPSS, Inc). Chi-2 test, Fisher’s exact test and multivariate analysis (logistic regression and Cox model) were performed in order to:

- Compare *KIT*^*L541*^ prevalence in GIST and in the normal donor series.
- Study the distribution of known prognostic factors (tumor size, mitotic index and organ site) according to the presence of the SNP.
- Investigate the possible correlation between *KIT*^*L541*^ and clinical and biological characteristics of GIST (disease stage, mutations, relapse).

Relapse free survival (RFS) and overall survival (OS) of patients harboring or not *KIT*^*L541*^ variant were plotted using the after Kaplan-Meier method and compared by log-rank test.

## Results

### KIT^L541^ and WT KIT have similar phosphorylation status in NIH3T3 cells

**Fig. 1:**
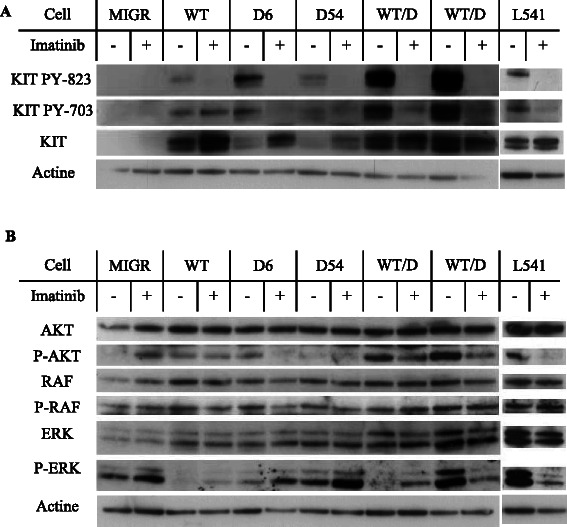
*KIT* maturation and activation status and related intracellular signaling in *KIT*-expressing cells treated or not with imatinib. Western blotting analysis after a 24-h exposure to 1 μM of imatinib of the expression and the phosphorylation of **a**. mature (145 kDa) and immature (125 kDa) forms of *KIT* on residues Tyr^703^ and Tyr^823^ and **b**. *AKT*, *RAF* and *ERK*1/2

KIT function and phosphorylation status was investigated using western blotting analyses (Fig. 1a). No basal expression or phosphorylation of KIT was detected in MIGR cell control. The two forms of the receptor, 125 kDa (immature) and 145 kDa (mature), were found expressed in WT transfected cells. The 145-kDa form was found phosphorylated on both Y-823 and Y-703, at a basal level. The L541 cell line (expressing the polymorphic form of KIT, *KIT*^*L541*^) displayed the same expression and phosphorylation pattern as WT cell line upon SCF stimulation. D6 and D54 transfected mutants showed an increased spontaneous phosphorylation status on both residues. The immature 125-kDa form was also phosphorylated on both tyrosines but not in cell lines with WT or L541. In cells transfected with WT and D6 or D54 mutated *KIT*, phosphorylation status of both Y-703 and Y-823 was found increased, in line with reported KIT activation status in tumors exhibiting homo/heterozygous mutations of the receptor [18]. Overall, in WT and L541 cell lines, only membrane mature KIT is phosphorylated upon SCF exposure, while both mature and immature KIT are constitutively activated in D6 and D54 cell lines. In all cell lines, Y-823 phosphorylation is completely lost after imatinib treatment.

### Intracellular signaling in KIT-expressing cell lines with or without imatinib treatment

KIT signaling pathways and the phosphorylation status of AKT (PI3K-AKT pathway), RAF, ERK1 and ERK2 (MAPK pathway) was assessed in these cell lines.

L541 cell line was found to activate AKT at a similar level than the D6 and D54 mutants. After imatinib exposure, P-AKT increased in MIGR cell line, remained stable in WT and D54 cell lines, and decreased in D6 and in L541 cells.

RAF phosphorylation was similar in WT, D6 and D54 cell lines and was not affected by imatinib exposure. In contrast, the downstream ERK proteins were found to display phosphorylation, varied according to KIT mutation status: high levels of spontaneous phosphorylation are detectable in MIGR control, D54, D54/WT and in L541 cell lines, in particular ERK2 (42 kDa). Lower levels are observed in D6 cell lines cells whereas it is undetectable in WT cell lines. Exposure to imatinib was found to increase ERK phosphorylation in WT, D6, D54 and WT/D6 cell lines but not in WT/D54 and L541 cell lines.

### Overall, in L541 cell line, KIT displays an activation pattern of AKT and ERK1 or 2 similar to that of the mutated forms of the receptors in presence of SCF. We conclude that KIT^L541^ share some of the biochemical properties of the mutated KIT receptors

The association between the presence of the variant *KIT*^*L541*^ and clinical presentation and prognostic in GIST patients was then investigated.

### Clinical and biological characteristics of the two GIST patients series

This retrospective case–control study was conducted on two different series: 87 resected primaries GIST untreated until recurrence and 22 metastatic at diagnosis (or locally advanced/unresectable) GIST. The characteristics of the two series of GIST are presented in Table 1.

**Table 1:** Baseline characteristics of the two retrospective series of GIST

Characteristics	First series of resected primary GIST	Second series of metastatic or locally advanced at diagnosis GIST
N	87	22
Male	42	13
Female	45	9
Age (years)	65 (22–84)	64 (39–79)
Median size (mm)	58 (17–400)	90 (45–240)
Location		
Stomach	52 (60 %)	8 (36 %)
Small bowel	24 (28 %)	11 (50 %)
Other locations	11 (12 %)	3 (14 %)
Mitotic index per 50 HPF		
≤ 5	51 (59 %)^a^	5 (26 %)^a^
6–10	12 (14 %)^a^	4 (21 %)^a^
> 10	23 (27 %)^a^	10 (52 %)^a^
Unknown	1	3
Mutation status		
KIT ex 11	40 (54 %)^a^	12 (75 %)^a^
KIT ex 9	5 (7 %)^a^	3 (19 %)^a^
KIT ex 13	1 (1 %)^a^	1 (6 %)^a^
KIT ex 17	2 (3 %)^a^	0
PDGFRA ex 18	11 (15 %)^a^	0
D842V	9	0
No mutation on *KIT* or *PDGFRA* hotspots	15 (20 %)^a^	0
Unknown	13	6
Prevalence of the variant *KIT*^L541^	15 (17 %)	9 (41 %)

^a^Percentages are determined on the evaluable cases

*HPF* high-power field

Within the first series, clinical and biological characteristics are similar to those described in literature, with a median age at diagnosis at 65 years old (range = 22–84), a sex ratio about 1 (42/45), a majority of gastric location (*n* = 52, 60 %) and a majority of *KIT* exon 11 mutation (*n* = 40, 54 %). With a median follow-up of 68 months, there were 17 (20 %) local or metastatic relapse at 5 years: 7 of 12 (59 %) *KIT* exon 11 mutated, 1 of 12 (8 %) *KIT* exon nine mutated, 1 of 12 (8 %) *KIT* exon 13 mutated, 2 of 12 (17 %) with mutations in *PDGFRA* (exon 18, D842V), 1 of 12 (8 %) WT and five with an undetermined mutational status.

As expected, tumor’s parameters of the second series (size, location and mitotic rate) were worse according to Miettinen classification: higher median size (90 mm, range 45–240, versus 58 mm, range 17–400), higher mitotic rate and more small bowel location (*n* = 11, 50 %, versus *n* = 24, 28 %).

In the control group of 60 healthy blood donors, the sex ratio was 1 (30/30) and the median age was 45 years old (range 19–63).

### Clinical and biological impact of the L541 KIT variant

Of all 109 patients, 24 (22 %) harboured *KIT*^*L541*^, similarly to the control group of healthy donors (*n* = 10 of 60, 17 %; Chi-squared test, *p* = 0.4). The variant was always detected in both GIST tumor cells and dry pellets PBMC when both types of samples were available (*n* = 15), confirming that it is not a mutation but a somatic genetic variant. Distribution of the clinical and biological characteristics of GIST harboring or not *KIT*^*L541*^ are detailed in Table 2, showing no differences between those 2 groups regarding size, the location, the mitotic index and the distribution of *KIT* and *PDGFRA* mutations. Importantly, prevalence of *KIT*^*L541*^ was statistically higher in this series of GIST patients metastatic at diagnosis vs non-metastatic patients at diagnosis (*n* = 9/22, 41 % versus *n* = 15/87, *n* = 17 %; Fischer’s exact test, *p* = 0.02).

**Table 2:** Clinical and biological characteristics of the two GIST patients series according to *KIT*^*L541*^

Clinical and biological characteristics	*KIT* ^L541^	*KIT* ^M541^	*P* ^*b*^
N	24	85	
Median size (mm)	50 (17–240)	60 (20–400)	
Location			
Stomach	12 (50 %)	48 (56 %)	0.6
Small bowel	7 (29 %)	28 (33 %)	1
Mitotic index			
≤ 5	11 (50 %)^a^	46 (54 %)	0.8
6–10	2 (9 %)^a^	15 (18 %)	0.5
> 10	9 (41 %)^a^	24 (28 %)	0.4
Mutation status			
*KIT* Mutations	15 (75 %)^a^	49 (70 %)^a^	0.8
*KIT* exon 11	10 (50 %)^a^	42 (60 %)^a^	0.5
*KIT* exon 9	3 (15 %)^a^	5 (7 %)^a^	0.4
*KIT* exon 13	2 (10 %)^a^	0	n/a
*KIT* exon 17	0	2 (3 %)^a^	n/a
*PDGFRA* Mutations	2 (10 %)^a^	9 (13 %)^a^	1
*PDGFRA* exon 18 D842V	1	8	
Wild Type	3 (15 %)^a^	12 (17 %)^a^	1
Undetermined	4	15	
Stage of the disease at diagnosis			
Localized	15 (62 %)	72 (85 %)	0.02
Metastatic (or locally advanced/unresectable)	9 (38 %)	13 (15 %)	

^a^Percentages are determined on the evaluable cases

^b^Chi-square test or Fisher’s exact test were applied as appropriate to check for differences according to the presence of *KIT*^*L541*^

**Fig. 2:**
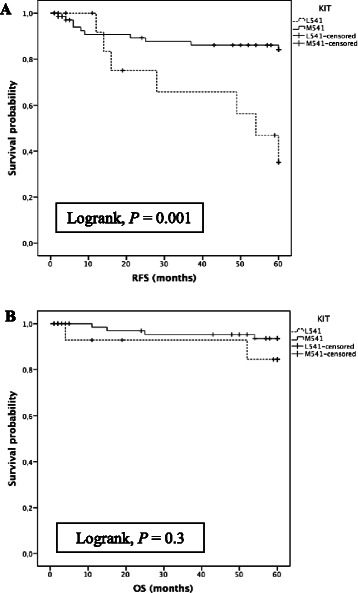
**a**. Kaplan–Meier analysis of RFS at 5 years of the first retrospective series of primary resected GIST stratified by *KIT*^*L541*^ status (*p* = 0.001); *x*-axis, time in months. **b**. Kaplan–Meier analysis of OS at 5 years of the first retrospective series of primary resected GIST stratified by *KIT*^*L541*^ status (*p* = 0.3); *x*-axis, time in months

In the first series of 87 non-metastatic patients, 15 had a *KIT*^*L541*^ (17 %). *KIT*^*L541*^ was significantly correlated to a higher rate of local or metastatic relapse at 5 years (*n* = 7/15, 47 %, versus *n* = 10/72, 14 %; Fisher’s exact test, *p* = 0.008), resulting in a lower relapse free survival (RFS) (Fig. 2a; Log-rank test, *p* =0.001).

Importantly, in multivariate analysis (*KIT*^*L541*^ status, tumor size, location, mitotic index and mutation status) using Cox model, *KIT*^*L541*^ was independently and directly associated to a lower RFS at 5 years (*p* = 0.004), together with tumor size (< or ≥ 50 mm, *p* = 0.03) and mitotic rate (< or ≥ 5 per 50 HPF, *p* = 0.006) (Table 3). L541 KIT was not correlated to the nature of the driver mutation.

**Table 3:** Impact of *KIT*^*L541*^, tumor size, location, mitotic index and mutational status on RFS at 5 years in multivariate analysis

Variable	HR (95 % CI)	*P*
*KIT* ^L541^	6.1 (1.8–21)	0.004
Tumor size (<50 mm versus ≥ 50 mm)	3.6 (1.1–12)	0.03
Location (gastric versus non gastric)	0.5 (0.1–2)	0.06
Mitotic index per 50 HPF (<5 versus ≥5)	6.2 (1.7–23)	0.006
Mutation status (all other versus KIT exon 11)	1.5 (0.4–5)	0.6

*HR* Hazard ratio, *CI* confidence intervals

Overall survival of the 2 groups was not significantly different (Fig. 2b; Log-rank test, *p* = 0.3). In the subgroup of WT and PDGFRA mutated GIST, *KIT*^*L541*^ was associated to a lower RFS at 5 years (Log-rank test, *p* = 0.02). In the subgroup of *KIT* mutated GIST, RFS of the 2 groups was not different although a trend was observed (Log-rank test, *p* = 0.09).

In the second series of 22 metastatic GIST at diagnosis, PFS and OS at 5 years of the patients harbouring or not *KIT*^*L541*^ were not different (Log-rank test, *p* = 0.2 and *p* = 0.6). Pooling this series of patients with all 24 relapsing patients from group 1 (17 relapses at 5 years and seven beyond 5 years) yield a group of 46 patients. In this pooled series as well, PFS and OS at 5 years of the metastatic patients receiving imatinib and harbouring or not *KIT*^*L541*^ were also not significantly different (Log-rank test, *p* = 0.6 and *p* = 0.6) indicating that *KIT*^*L541*^ is a prognostic factor for relapse but not a predictive factor for response to imatinib.

## Discussion

In this work, we investigated the biological and clinical significance of the SNP *KIT*^*L541*^ in GIST patients, its potential role in carcinogenesis and the potential predictive value of this variant for GIST patients outcome.

Using transfected cell lines, KIT^L541^ was found to exhibit a similar maturation and activation pattern than WT KIT but induced a mutant-like intracellular signalling in the presence of SCF. The role of the transmembrane domain in tyrosine kinase receptors dimerization and signalling is well known and mutations in this region can result in constitutive activation [22, 23]. The *in vitro* experiments of this study suggest that cells expressing KIT^L541^ may have a proliferative and survival advantage. The substitution of a methionine into a leucine in position 541, at the end of the transmembrane domain, seems to increase the ability of the receptor to activate *PI3K/AKT* and *MAPK* signalling pathways as compared with WT *KIT*.

The prevalence of this variant was investigated in two series of GIST patients and in a control group. *KIT*^*L541*^ was both detected in tumors and PBMCs DNA; it is a constitutional SNP variant unlike activating mutations of *KIT* and *PDGFRA*. We reported in this study a similar prevalence of *KIT*^*L541*^ in healthy donors and GIST patients. This SNP is not associated with specific activating mutations of *KIT* or *PDGFRA* and the proportion of *KIT*^*L541*^ does not differ between WT GIST and other. These results suggest that *KIT*^*L541*^ is not per se a risk factor for GIST development. However, *KIT*^*L541*^ was found associated with a higher risk of tumor progression and dissemination. In the first series of GIST patients, *KIT*^*L541*^ was associated with a higher risk of relapse, resulting in a lower relapse-free survival at 5 years. Importantly, this impact of *KIT*^*L541*^ was conserved in multivariate analysis using Cox model. Moreover, *KIT*^*L541*^ prevalence was higher in patients presenting with metastatic GIST at diagnosis. These results suggest that this variant is a prognostic factor for relapse and progression.

Conversely, *KIT*^*L541*^ was not found to be a predictive biomarker for the efficacy of imatinib in advanced phase. However, it must be noted that the power of the study remained limited by the small size of the cohort treated by imatinib, as well as subgroup analysis in subgroup of WT and PDGFRA mutated GIST (6 cases) could not be performed.

Sequence variations (SNP) of *KIT* have been described to play an important role in cancer. Rare SNP on *KIT* exon 10 (*A533D* and *F522C*) are identified in systemic aggressive mastocytosis [24] and lead to constitutive activation of the receptor. This variant (c.1621 A > C) in exon 10 of *KIT* (*KIT*^*L541*^) has previously been described in CML, AF, systemic mastocytosis and Merkel cell carcinoma. Inokuchi and al. [16] performed *in vitro* experiments on *KIT*^L541^ Ba/F3 cells, showing that tyrosine kinase activation and proliferative response of *KIT*^L541^ cells were slightly higher than WT *KIT* in medium containing 0.1 ng/ml SCF. Moreover, in a cohort of 80 patients with CML, *KIT*^*L541*^ was associated to a statistically lower median overall survival [16]. In vitro studies of Foster et al. [17] demonstrated that FDC-P1 cells transfected with *KIT*^L541^ showed an enhanced proliferative response to low levels of stem cell factor (SCF) (≤6.25 ng/ml). Grabellus et al. [25] showed that prevalence of *KIT*^*L541*^ in aggressive fibromatosis did not differ from the general population, suggesting that this variant was not involved in carcinogenesis of these tumors. In 2006, Gonçalves et al. [13] published the case of a major tumor response of imatinib, corresponding to a desmoid tumor expressing *KIT*^*L541*^. Conversely, Dufresne et al. [15] did not observe a predictive value of *KIT*^*L541*^ for response to imatinib for patients with AF. Currently, the significance of the *KIT*^*L541*^ variant in AF remains unclear. More recently, Iurlo et al. [18] suggest that the presence of *KIT*^*L541*^ in chronic eosinophilic leukemia could be a predictive factor of imatinib sensitivity.

Currently, GIST recurrence potential after complete surgery resection is measured using a set of predictive parameters: tumor size, mitotic rate, organ site, tumor rupture and nature of the driver mutation. These biomarkers still remain imperfect to identify accurately patients who will relapse and may be candidate to adjuvant imatinib treatment. Additional factors such as germline genetic variations and epigenetic phenomena (DNA hypomethylation and hypermethylation, microRNA alterations, etc.…) were found to be associated with GIST patient outcome [2, 8–12]. O’Brien et al. [26, 27] investigated the role of inherited genetic SNP in tumor development and have identified several SNP associated with GIST mutation subtypes (for example, CYP1B1 SNP and, as expected, deletion at *KIT* exon 11 codons 557–8). Besides, a few germline genetic variants of receptor tyrosine kinase (IGF1R, VEGFR) were previously found to be associated with clinical outcome [2, 9–12], suggesting the importance of somatic genome in GIST. Results of Angelini et al. [11] suggest that polymorphisms in the genes coding folate-metabolising enzymes may modify the risk of GIST and clinical outcome and results of Haller et al. [28] suggest SPP1 promoter methylation as a novel and independent prognostic parameter in GIST. *KIT*^*L541*^ belongs to this novel class of predictor of the risk of relapse in patients with GIST. Its detection could be useful for clinical management of GIST patients. However, the way to use those additional prognostic markers in routine remains unclear and further investigations are necessary.

## Conclusion

In conclusion, the variant *KIT*^*L541*^ was not found associated with increased risk of development of GIST but represents a predictive factor for relapse in localized GIST. Conversely, *KIT*^L541^ is not a predictive factor for response to imatinib in patients with GIST.

## Additional file

Additional file 1: Table S1. Characteristics of currently used antibodies. (DOC 33 kb)

## Abbreviations

AF: Aggressive fibromatosis
CAST-PCR: Competitive allele specific Taqman – PCR
CML: Chronic myelogenous leukemia
CPP: Comités de protection des personnes
Ct: Cycle threshold
FFPE: Formalin fixed paraffin embedded
ICF: Informed consent form
GIST: Gastrointestinal stromal tumors
MCSF: Macrophage colony-stimulating factor
MCSFR: Macrophage colony-stimulating factor receptor
OS: Overall survival
PBMC: Peripheral blood mononuclear cells
PCR: Polymerase chain reaction
PDGF: Pateled derived growth factor
PDGFR: Pateled derived growth factor receptor
RFS: Relapse free survival
SCF: Stem cell factor
SNP: Single nucleotide polymorphism

## References

[1] Cassier PA, Ducimetière F, Lurkin A, Ranchère-Vince D, Scoazec J-Y, Bringuier P-P (2010). A prospective epidemiological study of new incident GISTs during two consecutive years in Rhône Alpes region: incidence and molecular distribution of GIST in a European region. Br J Cancer.

[2] Angelini S, Ravegnini G, Fletcher JA, Maffei F, Hrelia P (2013). Clinical relevance of pharmacogenetics in gastrointestinal stromal tumor treatment in the era of personalized therapy. Pharmacogenomics.

[3] Cioffi A, Maki RG (2015). GI stromal tumors: 15 years of lessons from a rare cancer. J Clin Oncol Off J Am Soc Clin Oncol.

[4] Corless CL (2014). Gastrointestinal stromal tumors: what do we know now?. Mod Pathol.

[5] Lee J-H, Kim Y, Choi J-W, Kim Y-S (2013). Correlation of imatinib resistance with the mutational status of KIT and PDGFRA genes in gastrointestinal stromal tumors: a meta-analysis. J Gastrointest Liver Dis.

[6] Heinrich MC, Corless CL, Blanke CD, Demetri GD, Joensuu H, Roberts PJ (2006). Molecular correlates of imatinib resistance in gastrointestinal stromal tumors. J Clin Oncol Off J Am Soc Clin Oncol.

[7] ESMO/European Sarcoma Network Working Group (2014). Gastrointestinal stromal tumours: ESMO clinical practice guidelines for diagnosis, treatment and follow-up. Ann Oncol.

[8] Sioulas AD, Vasilatou D, Pappa V, Dimitriadis G, Triantafyllou K (2013). Epigenetics in gastrointestinal stromal tumors: clinical implications and potential therapeutic perspectives. Dig Dis Sci.

[9] Kwon O, Chung HY, Yu W, Bae HI, Chae YS, Kim JG (2012). Clinical significance of insulin-like growth factor gene polymorphisms with survival in patients with gastrointestinal stromal tumors. J Korean Surg Soc.

[10] Kang BW, Kim JG, Chae YS, Bae HI, Kwon O, Chung HY (2014). Clinical significance of vascular endothelial growth factor and vascular endothelial growth factor receptor-2 gene polymorphisms in patients with gastrointestinal stromal tumors. Asia Pac J Clin Oncol.

[11] Angelini S, Ravegnini G, Nannini M, Bermejo JL, Musti M, Pantaleo MA (2015). Folate-related polymorphisms in gastrointestinal stromal tumours: susceptibility and correlation with tumour characteristics and clinical outcome. Eur J Hum Genet.

[12] Ravegnini G, Nannini M, Sammarini G, Astolfi A, Biasco G, Pantaleo MA (2015). Personalized medicine in Gastrointestinal Stromal Tumor (GIST): Clinical implications of the somatic and germline DNA analysis. Int J Mol Sci.

[13] Gonçalves A, Monges G, Yang Y, Palmerini F, Dubreuil P, Noguchi T (2006). Response of a KIT-positive extra-abdominal fibromatosis to imatinib mesylate and KIT genetic analysis. J Natl Cancer Inst.

[14] Dufresne A, Bertucci F, Penel N, Le Cesne A, Bui B, Tubiana-Hulin M (2010). Identification of biological factors predictive of response to imatinib mesylate in aggressive fibromatosis. Br J Cancer.

[15] Dufresne A, Alberti L, Brahmi M, Kabani S, Philippon H, Pérol D (2014). Impact of KIT exon 10 M541L allelic variant on the response to imatinib in aggressive fibromatosis: analysis of the desminib series by competitive allele specific Taqman PCR technology. BMC Cancer.

[16] Inokuchi K, Yamaguchi H, Tarusawa M, Futaki M, Hanawa H, Tanosaki S (2002). Abnormality of c-kit oncoprotein in certain patients with chronic myelogenous leukemia--potential clinical significance. Leukemia.

[17] Foster R, Byrnes E, Meldrum C, Griffith R, Ross G, Upjohn E (2008). Association of paediatric mastocytosis with a polymorphism resulting in an amino acid substitution (M541L) in the transmembrane domain of c-KIT. Br J Dermatol.

[18] Iurlo A, Gianelli U, Beghini A, Spinelli O, Orofino N, Lazzaroni F (2014). Identification of kit(M541L) somatic mutation in chronic eosinophilic leukemia, not otherwise specified and its implication in low-dose imatinib response. Oncotarget.

[19] Tabone-Eglinger S, Subra F, El Sayadi H, Alberti L, Tabone E, Michot J-P (2008). KIT mutations induce intracellular retention and activation of an immature form of the KIT protein in gastrointestinal stromal tumors. Clin Cancer Res.

[20] Fayet Y, Chasles V, Ducimetière F, Collard O, Berger C, Meeus P (2014). To answer rare cancer issues. Geographical analysis of EMS sarcoma cohort in the Rhône-Alpes region. Bull Cancer.

[21] Blay J-Y, Le Cesne A, Ray-Coquard I, Bui B, Duffaud F, Delbaldo C (2007). Prospective multicentric randomized phase III study of imatinib in patients with advanced gastrointestinal stromal tumors comparing interruption versus continuation of treatment beyond 1 year: the French Sarcoma Group. J Clin Oncol Off J Am Soc Clin Oncol.

[22] Webster MK, Donoghue DJ (1996). Constitutive activation of fibroblast growth factor receptor 3 by the transmembrane domain point mutation found in achondroplasia. EMBO J.

[23] Weiner DB, Liu J, Cohen JA, Williams WV, Greene MI (1989). A point mutation in the neu oncogene mimics ligand induction of receptor aggregation. Nature.

[24] Orfao A, Garcia-Montero AC, Sanchez L, Escribano L, REMA (2007). Recent advances in the understanding of mastocytosis: the role of KIT mutations. Br J Haematol.

[25] Grabellus F, Worm K, Sheu S-Y, Siffert W, Schmid KW, Bachmann HS (2011). The prevalence of the c-kit exon 10 variant, M541L, in aggressive fibromatosis does not differ from the general population. J Clin Pathol.

[26] O’Brien KM, Orlow I, Antonescu CR, Ballman K, McCall L, DeMatteo R (2013). Gastrointestinal stromal tumors, somatic mutations and candidate genetic risk variants. PLoS ONE.

[27] O’Brien KM, Orlow I, Antonescu CR, Ballman K, McCall L, Dematteo R (2013). Gastrointestinal stromal tumors: a case-only analysis of single nucleotide polymorphisms and somatic mutations. Clin Sarcoma Res.

[28] Haller F, Zhang JD, Moskalev EA, Braun A, Otto C, Geddert H (2015). Combined DNA methylation and gene expression profiling in gastrointestinal stromal tumors reveals hypomethylation of SPP1 as an independent prognostic factor. Int J Cancer J Int Cancer.

