# Supplementary material for: KIT exon 10 variant (c.1621 A > C) single nucleotide polymorphism as predictor of GIST patient outcome
Source: BMC Cancer. 2015 Oct 24;15:780. doi: 10.1186/s12885-015-1817-5 (PMC4619434; doi:10.1186/s12885-015-1817-5)
Supplement: Additional file 1: Table S1. — Characteristics of currently used antibodies. (DOC 33 kb) [file 12885_2015_1817_MOESM1_ESM.doc]

**Supplementary Material 1.** Characteristics of currently used antibodies.

| **Antibody** | **Molecular weight (kDa)** | **Dilution** | **Provided by** |
| --- | --- | --- | --- |
| λ c-kit | 125/145 | 1/1000 | Dako |
| λ PY703 c-kit | 125/145 | 1/1000 | Biosource |
| λ PY823 c-kit | 125/145 | 1/1000 | Biosource |
| λ AKT | 60 | 1/1000 | Santa Cruz |
| λ P-AKT | 60 | 1/500 | Cell signalling |
| λ Raf | 74 | 1/500 | Santa Cruz |
| λ P-Raf | 74 | 1/500 | Cell signalling |
| λ Erk | 42/44 | 1/1000 | Cell signaling |
| λ P-Erk (T202, Y204) | 42/44 | 1/1000 | Cell signalling |
| λ β-actin | 42 | 1/2000 | SIGMA |
| λ Rabbit | n/a | 1/100000 | Immunotech |
| λ Mouse | n/a | 1/100000 | Immunotech |

n/a, no answer.
